# Predicting preoperative axillary lymph node metastasis to guide surgical decisions in invasive breast cancer

**DOI:** 10.3389/fonc.2026.1786051

**Published:** 2026-05-29

**Authors:** Qi Xin, Zhilin Yang

**Affiliations:** 1 Department of Emergency Surgery, Shaanxi Provincial People’s Hospital, Xi’an, China; 2 Department of Intensive Care Unit (ICU), The Second Clinical College of Shanxi Medical University, Taiyuan, China

**Keywords:** ALNM, clinicopathological indicators, invasive breast cancer, nomogram, serological indicators, ultrasound indicators

## Abstract

**Background:**

In the clinical management of patients with invasive breast cancer (IBC), the precise identification of axillary lymph node metastasis (ALNM) is of paramount importance for guiding axillary surgery and formulating corresponding treatment strategies. Currently, the clinical need to accurately predict the risk of ALNM in patients with IBC, so as to determine whether axillary surgery (including sentinel lymph node biopsy) can be safely omitted, has not been fully met.

**Methods:**

The clinical data of 454 patients with IBC were retrospectively analyzed, and the patients were randomly divided into a training group and a validation group at a ratio of 3:1. Independent predictors related to ALNM were identified through univariate and multivariate Logistic regression analysis. Accordingly, a nomogram model integrating clinicopathological, ultrasound and serological indicators along with additional clinical parameters was constructed and validated by receiver operating characteristic (ROC) curve analysis, calibration plots, and decision curve analysis (DCA). The primary outcome measure was the incidence of ALNM.

**Results:**

The ALNM rates were 31.4% and 27.4% in the training and verification groups, respectively. Multivariate regression analysis indicated that tumor size, circumscribe margin, ultrasonic lymph node status, WBC, ER, and BI-RADS were independent risk factors for ALNM in patients with IBC. In both the training group and the validation group, the nomogram exhibited modest predictive performance (training group AUC = 0.741; validation group AUC = 0.705). The Ultrasonic lymph node status (normal vs. abnormal) alone yielded an AUC of 0.620 in the training cohort and 0.627 in the validation cohort for predicting ALNM. In comparison, our nomogram achieved significantly higher AUCs (training: 0.741, P = 0.0017; validation: 0.705, P = 0.1772; DeLong test). Additionally, the nomogram demonstrated satisfactory calibration and clinical utility as evidenced by the calibration curve and DCA.

**Conclusion:**

By integrating clinicopathological, ultrasonic and serological indicators, this nomogram can enhance the accuracy of predicting ALNM in patients with IBC. However, given its moderate discriminative ability, the model can support preoperative risk stratification but cannot replace standard axillary staging procedures (including sentinel lymph node biopsy). Our findings provide a basis for decision-making regarding individualized axillary lymph node surgery.

## Introduction

Breast cancer is the most common malignant tumor in women worldwide, and in recent years, with the advancement of treatment, the survival rate of patients has improved significantly (1). However, recurrence and distant metastasis remain the leading causes of death in patients. In patients with IBC, ALNs are the main route for breast cancer cell metastasis, and approximately 70% of breast lymph fluid passes through the axillary area. Therefore, the status of ALNs is a key factor in assessing cancer staging and developing treatment plans, and early identification of ALNM is essential for treatment (2, 3).

Currently, sentinel lymph node biopsy (SLNB) has become the standard of care for axillary staging in clinically node-negative breast cancer patients, as recommended by major guidelines including the National Comprehensive Cancer Network (NCCN) and European Society for Medical Oncology (ESMO) (4, 5). Compared with axillary lymph node dissection (ALND), SLNB offers comparable survival outcomes while significantly reducing surgical morbidity (6). A recent meta-analysis of seven randomized controlled trials demonstrated that SLNB is associated with a 65% lower risk of lymphedema compared with ALND, with no significant differences in 5-, 8-, or 10-year overall survival, disease-free survival, or recurrence rates (6). However, SLNB is not without limitations. The procedure carries a false-negative rate of approximately 5-10% in clinically node-negative patients, which can be reduced to below 10% through dual-tracer mapping techniques and removal of three or more sentinel nodes (7–9). Potential complications, though substantially less frequent than with ALND, include temporary sensory disturbances (reported in approximately 10% of patients), transient shoulder mobility restriction (approximately 7%), and mild lymphedema (approximately 7-8%) (7). These considerations underscore the clinical need for accurate preoperative prediction of axillary lymph node metastasis to optimize patient selection for SLNB and potentially avoid unnecessary axillary surgery altogether.

With the continuous advancement of medical technology, it is particularly urgent to develop non-invasive methods to predict ALNM in IBC patients. As a routine screening method for breast cancer, breast ultrasound not only performs well in evaluating the size and location of tumors and delineating the internal structure of lymph nodes, but also shows great potential in diagnosing early metastatic lesions (10). Compared with MRI or other radiomics techniques, breast ultrasound is a non-invasive diagnostic method with low cost, convenient operation, strong repeatability, and no radiation (11). Some studies have also confirmed that the clinicopathological characteristics, including age, tumor size, location, histological grade, and receptor status, are key factors in predicting axillary lymph node metastasis (12–14). In recent years, indicators such as serum markers and blood cell counts have also been found to be closely related to the metastasis, prognosis and treatment effect of malignant tumors, and the acquisition of these indicators is relatively easy and less invasive (15). However, the relationship between serological indicators and ALNM is still limited, and the exact mechanism needs to be further clarified.

Given the high heterogeneity of IBC, different patients may have different disease progression and prognosis. Although several predictive models for ALNM have been developed, most are based on Western populations with limited applicability to Chinese patients, rely on a single category of predictors (e.g., ultrasound alone or clinicopathological features alone), or require complex radiomics or genetic testing that hinders clinical adoption. More importantly, in the current era of axillary surgical de−escalation, where landmark trials such as SOUND and INSEMA have demonstrated that sentinel lymph node biopsy can be safely omitted in selected low−risk patients, the clinical need has shifted from simply predicting ALNM to accurately identifying patients with a sufficiently low risk of metastasis to safely forgo any axillary surgery. To address these gaps, we developed a practical nomogram that integrates widely accessible clinicopathological, ultrasound, and serological indicators (including white blood cell count, which has rarely been incorporated into such models) to preoperatively predict ALNM risk in a Chinese cohort. Our model is designed to support individualized axillary de−escalation decisions in routine clinical practice.

## Materials and methods

### Patients

This study included 454 patients with IBC diagnosed in the Second Clinical College of Shanxi Medical University from January 2020 to July 2024, which constituted the main study cohort. Exclusion criteria included: patients who had received preoperative treatment such as neoadjuvant chemotherapy; Patients with a history of other malignancies, immune system or blood system disorders; And patients with multifocal, bilateral, recurrent, metastatic, or other pathological types of breast cancer. Patients were randomly allocated to a training set and a validation set in a 3:1 ratio using a fixed random seed (set.seed(123) in R version 4.1.3) to ensure reproducibility. This retrospective study follows the ethical guidelines of the Declaration of Helsinki and has been approved by the Ethics Committee of the Second Clinical Hospital of Shanxi Medical University. Considering that the study will not adversely affect the rights and health of the participants and that the level of risk does not exceed the minimum risk, the procedure of informed consent is exempted. In addition, patient privacy and personally identifiable information are properly protected.

### Definitions

For the diagnosis of lymph node metastasis, the following criteria were employed: Positive axillary lymph node (ALN): Positive outcomes are affirmed through axillary lymph node dissection and needle biopsy; Axillary lymph node (ALN) negative: A negative finding is verified by axillary lymph node dissection and sentinel lymph node biopsy. In patients with negative sentinel lymph node biopsy (SLND) results, axillary lymph nodes are regarded as free of metastasis. Based on these criteria, patients were classified into those with axillary lymph node metastasis and those without.

### Data collection

In this study, we collected the following clinical and laboratory indicators from patients:

(1) Demographic characteristics: age and menstrual status. (2) Pathological indicators: androgen receptor (AR), estrogen receptor (ER), progesterone receptor (PR), human epidermal growth factor receptor 2 (HER2), P53, Ki67. (3) Ultrasound indicators: tumor location, size, margin, morphology, color Doppler flow imaging (CDFI) characteristics, axillary lymph node status, and breast Imaging Reporting and Data System (BI-RADS) class. (4) Serological indicators: alanine aminotransferase, aspartate aminotransferase, total bilirubin, urea, creatinine, D dimer, white blood cell count, red blood cell count, platelet count, platelet volume distribution width, neutrophil absolute value, lymphocyte absolute value, monocyte absolute value, eosinophils absolute value, basophils absolute value.

For ultrasound evaluation, the following definitions were used: Tumor size was measured as the maximum diameter (in cm) of the primary tumor on gray−scale ultrasound. Circumscribe margin was classified as “clear” (well−defined, sharp border between tumor and surrounding tissue) or “vague” (indistinct, irregular, or spiculated border). Tumor morphology was categorized as “regular” (oval or round shape) or “irregular” (lobular, spiculated, or ill−defined shape). CDFI (color Doppler flow imaging) was graded as “no abnormality” (absence of blood flow signal within the tumor) or “positive” (presence of any detectable blood flow signal). Axillary lymph node status was considered abnormal if any of the following criteria were met: (1) round or oval lymph node shape; (2) absent or displaced echogenic hilum; (3) minimal nodal diameter >5 mm; (4) irregular cortical thickening >2 mm. Otherwise, the axillary lymph node was classified as normal. BI-RADS category was assigned according to the American College of Radiology Breast Imaging Reporting and Data System (5th edition) (16).

### Statistical analysis

The final cohort of patients was randomly allocated into a training group and a validation group in a 3:1 ratio using a fixed random seed (set.seed(123) in R version 4.1.3) to ensure reproducibility. Continuous variables adhering to a normal distribution were expressed as mean ± standard deviation, whereas non-normally distributed continuous variables were presented as median (interquartile range). Categorical variables were reported as percentages. Prior to multivariable logistic regression, we assessed multicollinearity among the independent variables using the variance inflation factor (VIF). A VIF > 5 was considered indicative of significant multicollinearity. Continuous variables, including tumor size and WBC count were entered into the model as continuous measurements without categorization. Logistic regression analysis was employed to identify potential risk factors for LNM, and a nomogram was subsequently constructed using multivariate logistic regression analysis. The Receiver Operating Characteristic (ROC) curve was utilized to validate the nomogram model and evaluate its accuracy in predicting LNM. Calibration curves were employed to assess the concordance between observed outcomes and predicted probabilities. Additionally, Decision Curve Analysis (DCA) was conducted to determine the clinical net benefit of the predictive model.

Statistical analyzes were performed using R version 4.1.3, with a significance threshold set at *P* < 0.05.

## Results

### Basic characteristics

**Figure 1:**
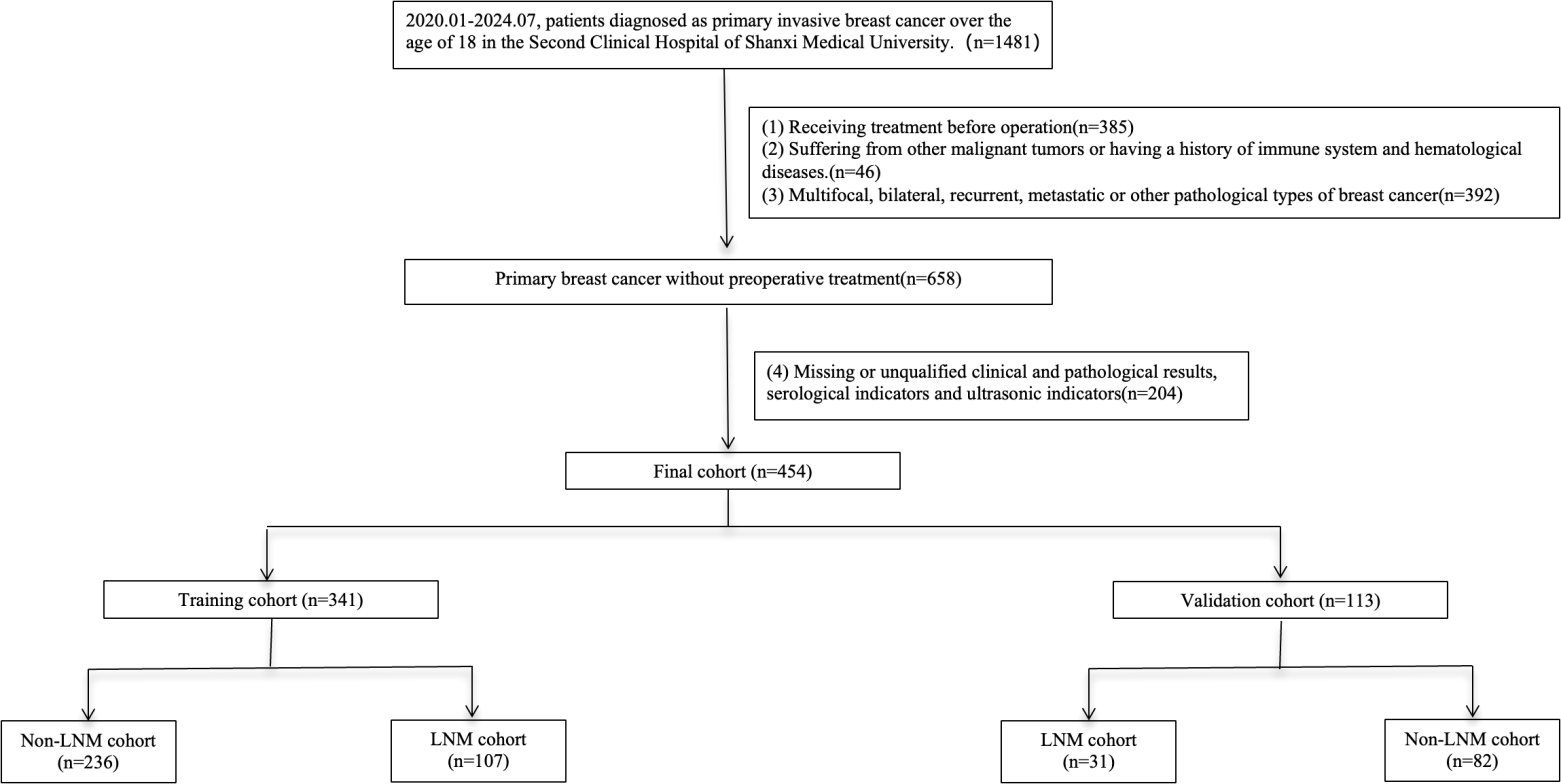
The flowchart of patient selection from the Shanxi Hospital.

The patient’s flow chart is shown in **Figure 1**. The study included 454 patients with invasive breast cancer, with a median age of 54.5 years. The clinical characteristics of these eligible patients are shown in **Table 1**. Baseline clinical data were comparable between the training and validation groups, and among all participants, 138 patients with invasive breast cases developed axillary lymph node metastases.

**Table 1:** Baseline characteristics of participants.

Variable	Total (n =454)	Training (n=341)	Validation (n=113)	*P* value
LNM, n (%)				0.502
No	318 (70.0)	236 (68.6)	82 (72.6)	
Yes	138 (30.4)	107 (31.4)	31 (27.4)	
Age (years)	54 (46,63)	55 (46,63)	52 (46,62)	0.361
Menopause state, n (%)				0.434
Premenopausal	340 (74.8%)	259 (76%)	81 (71.7%)	
Postmenopausal	114 (25.1%)	82 (24%)	32 (28.3%)	
AR (%)	90 (60,90)	90 (65,90)	90 (30,90)	0.387
ER (%)	90 (60,90)	90 (5,90)	90 (0,90)	0.463
PR (%)	30 (0,80)	30 (0,80)	40 (0,90)	0.747
HER2 state, n (%)				0.372
negative	267 (58.8%)	196 (57.5%)	71 (62.8%)	
positive	187 (41.1%)	145 (42.5%)	42 (37.2%)	
P53 (%)	15 (15,30)	15 (15,30)	15 (15,30)	0.295
Ki67 (%)	30 (15,50)	30 (15,50)	30 (19,60)	0.303
Tumor size (cm²)	2.23 (1.03,3.64)	2.28 (1.01,3.65)	2.11 (1.04,3.75)	0.962
Circumscribe margin, n (%)				0.990
Vague	279 (61.4%)	209 (61.3%)	70 (61.9%)	
Clear	175 (38.5%)	132 (38.7%)	43 (38.1%)	
Tumor morphology, n (%)				0.875
regular	48 (10.5%)	37 (10.9%)	11 (9.7%)	
irregular	406 (89.4%)	304 (89.1%)	102 (90.3%)	
CDF1, n (%)				0.088
No abnormality	153 (33.7%)	107 (31.4%)	46 (40.7%)	
Blood flow signal positive	301 (66.2%)	234 (68.6%)	67 (59.3%)	
Ultrasonic lymph node status, n (%)				0.104
Normal	213 (46.9%)	152 (44.6%)	61 (54%)	
Abnormal	241 (53%)	189 (55.4%)	52 (46%)	
Tumor location, n (%)				0.543
Outer upper quadrant	191 (42%)	144 (42.2%)	47 (41.6%)	
Outer lower quadrant	35 (7.7%)	27 (7.9%)	8 (7.1%)	
Inner lower quadrant	72 (15.8%)	58 (17%)	14 (12.4%)	
Inner upper quadrant	156 (34.4%)	112 (32.8%)	44 (38.9%)	
BI-RADS, n (%)				0.975
Level 2	1 (0.2%)	1 (0.3%)	0 (0%)	
Level 3	19 (4.1%)	14 (4.1%)	5 (4.4%)	
Level 4	349 (76.8%)	263 (77.1%)	86 (76.1%)	
Level 5	70 (15.4%)	52 (15.2%)	18 (15.9%)	
Level 6	15 (3.3%)	11 (3.2%)	4 (3.5%)	
ALT (U/L)	16.5 (12.5,23.4)	16.3 (12.7,23.6)	17.9 (11.8,23.2)	0.898
AST (U/L)	20.7 (17.6,24.8)	20.7 (17.6,25.0)	20.5 (17.4,24.0)	0.459
TBIL (umol/L)	11.9 (9.5,15.5)	12.0 (9.5,15.7)	11.7 (9.6,15.1)	0.863
UREA (mmol/L)	4.8 (4.0,5.6)	4.8 (4.1,5.7)	4.7 (3.9,5.5)	0.322
CREA (umol/L)	56 (51,62)	56 (51,62)	55 (50,63)	0.629
GLU (mmol/L)	5.21 (4.69,5.93)	5.23 (4.67,5.97)	5.14 (4.73,5.90)	0.742
D-dimer (ng/mL)	87.5 (57.8,143.0)	91 (57,139)	83 (58.5,148)	0.883
WBC (/L)	5.82 (4.88,6.85)	5.87 (4.90,6.88)	5.76 (4.82,6.73)	0.646
RBC (/L)	4.48 (4.20,4.73)	4.48 (4.19,4.71)	4.47 (4.23,4.81)	0.358
PLT (/L)	245 (197,287)	244 (198,287)	247 (196,291)	0.841
PDW (fL)	14.8 (11.7,16.0)	15.0 (11.7,16.0)	14.2 (11.5,16.0)	0.731
NEU (/L)	3.54 (2.83,4.37)	3.54 (2.81,4.37)	3.53 (2.93,4.41)	0.841
LYM (/L)	1.70 (1.38,2.13)	1.72 (1.40,2.14)	1.68 (1.30,2.13)	0.355
MON (/L)	0.36 (0.28,0.45)	0.37 ( (0.28,0.46)	0.36 (0.28,0.44)	0.325
EOS (/L)	0.07 (0.04,0.12)	0.07 (0.04,0.12)	0.07 (0.04,0.12)	0.818
BASO (/L)	0.03 (0.02,0.04)	0.03 (0.02,0.04)	0.03 (0.02,0.04)	0.351

LYM, Lymph Node Metastasis; AR, Androgen Receptor; ER, Estrogen Receptor; PR, Progesterone Receptor; HER2, Human Epidermal Growth Factor Receptor 2; P53, Tumor Protein P53; Ki-67, A proliferation marker in cancer; CDFI, Color Doppler Flow Imaging; BI-RADS, Breast Imaging Reporting and Data System; ALT, Alanine Aminotransferase; AST, Aspartate Aminotransferase; TBIL, Total Bilirubin; UREA, Urea; CREA, Creatinine; GLU, Glucose; D-dimer, A protein fragment found in the blood; WBC: White Blood Cell count; RBC, Red Blood Cell count; PLT, Platelet count; PDW, Platelet Distribution Width; NEU, Neutrophil count; LYM, Lymphocyte count; MON, Monocyte count; EOS, Eosinophil count; BASO, Basophil count.

### Development of nomogram

**Figure 2:**
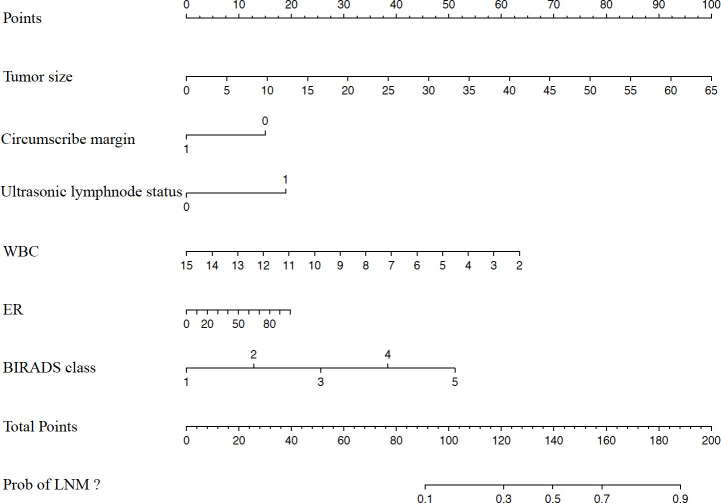
Nomogram to estimate the risk of LNM in patients with breast cancer. Variable assignments: ER (estrogen receptor, % positive, continuous); Tumor size (cm, continuous); Circumscribe margin (0 = vague, 1 = clear); Ultrasonic lymph node status (0 = normal, 1 = abnormal); BI-RADS class (continuous scale from 2 to 6); WBC (white blood cell count, ×10^9^/L, continuous). BI-RADS, Breast Imaging Reporting and Data System; WBC, White Blood Cell count; ER, Estrogen Receptor.

Univariate logistic regression analysis identified 8 variables that were significantly associated with LNM, including ER, CDFI, BI-RADS, WBC, BE, Ultrasonic lymph node status, Tumor size, Circumscribe margin, Tumor morphology (**Table 2**). Multicollinearity diagnostics revealed no significant collinearity among the six independent variables included in the final multivariable model (all VIF values < 2.5). A multivariate regression analysis of the training set, utilizing the significantly different variables, indicated that ER, BI-RADS, WBC, Tumor size, circumscribe margin and Ultrasonic lymph node status were identified as independent risk factors for the development of LNM in patients with IBC (**Table 3**). Based on these variables, a nomogram was constructed to predict LNM in invasive breast cancer patients (**Figure 2**). In the nomogram, the following variable assignments were used: circumscribe margin (0 = vague, 1 = clear), ultrasonic lymph node status (0 = normal, 1 = abnormal), and BI-RADS class (2 to 6, treated as continuous). ER, tumor size, and WBC were entered as continuous measurements.

**Table 2:** Univariate analysis of predictive variables of LNM in the training cohort.

Variables	OR	95% CI	*P* value
Age (years)	0.997	0.978-1.016	0.740
Menopause state	0.947	0.553-1.622	0.842
AR	1.002	0.995-1.009	0.529
ER	1.008	1.002-1.014	0.013
PR	1.004	0.998-1.010	0.250
HER2 state	0.906	0.612-1.545	0.906
P53	0.991	0.975-1.007	0.289
Ki67	1.001	0.992-1.010	0.850
Tumor size(cm²)	1.121	1.035-1.213	0.005
Circumscribe margin	0.443	0.268-0.732	0.001
Tumor morphology	3.232	1.222-8.543	0.018
CDF1	2.204	1.285-3.779	0.004
Ultrasonic lymph node status	2.796	1.707-4.581	<0.001
Tumor location	1.179	0.949,1.464	0.137
BI-RADS	2.400	1.480-3.892	<0.001
ALT(U/L)	0.998	0.972-1.017	0.870
AST(U/L)	1.005	0.980-1.030	0.715
TBIL (umol/L)	0.978	0.936-1.022	0.317
UREA (mmol/L)	0.905	0.759-1.080	0.270
CREA (umol/L)	1.001	0.977-1.026	0.921
GLU (mmol/L)	1.111	0.974-1.268	0.117
D-dimer(ng/mL)	1.002	1.000-1.004	0.100
WBC(/L)	0.867	0.753-0.997	0.046
RBC(/L)	1.018	0.976-1.061	0.413
PLT(/L)	0.998	0.995-1.002	0.321
PDW (fL)	0.938	0.857-1.026	0.161
NEU(/L)	0.198	0.754-1.060	0.198
LYM(/L)	1.003	0.910-1.104	0.957
MON(/L)	1.094	0.836-1.430	0.513
EOS(/L)	0.952	0.346-2.614	0.923
BASO(/L)	178.957	0.056-570501.761	0.208

AR, Androgen Receptor; ER, Estrogen Receptor; PR, Progesterone Receptor; HER2, Human Epidermal Growth Factor Receptor 2; P53, Tumor Protein P53; Ki-67, A proliferation marker in cancer; CDFI, Color Doppler Flow Imaging; BI-RADS, Breast Imaging Reporting and Data System; ALT, Alanine Aminotransferase; AST, Aspartate Aminotransferase; TBIL, Total Bilirubin; UREA, Urea; CREA, Creatinine; GLU, Glucose; D-dimer, A protein fragment found in the blood; WBC: White Blood Cell count; RBC, Red Blood Cell count; PLT, Platelet count; PDW, Platelet Distribution Width; NEU, Neutrophil count; LYM, Lymphocyte count; MON, Monocyte count; EOS, Eosinophil count; BASO, Basophil count.

**Table 3:** Multivariate logistic regression analysis of independent predictors of LNM in the training cohort.

Variables	β	SE	Wald	P-value	OR (95% Cl)
ER	0.009	0.003	6.763	0.009	1.009 (1.002-1.016)
Tumor size	0.070	0.033	4.362	0.037	1.072 (1.004-1.144)
Circumscribe margin	-0.677	0.274	6.095	0.014	0.508 (0.297-0.870)
Ultrasonic lymph node status	0.853	0.271	9.878	0.002	2.346 (1.379-3.994)
BI-RADS class	0.578	0.230	6.320	0.012	1.782 (1.136-2.797)
WBC	-0.221	0.083	7.061	0.008	0.802 (0.682-0.944)
Constant	-2.902	1.062	7.459	<0.05	——

Continuous variables (tumor size, WBC) were entered as continuous measurements. VIF values for all variables were < 2.5, indicating no significant multicollinearity. For categorical variables: circumscribe margin (0 = vague, 1 = clear); ultrasonic lymph node status (0 = normal, 1 = abnormal).

BI-RADS, Breast Imaging Reporting and Data System; WBC, White Blood Cell count; ER, Estrogen Receptor.

### Verification of nomogram

**Figure 3:**
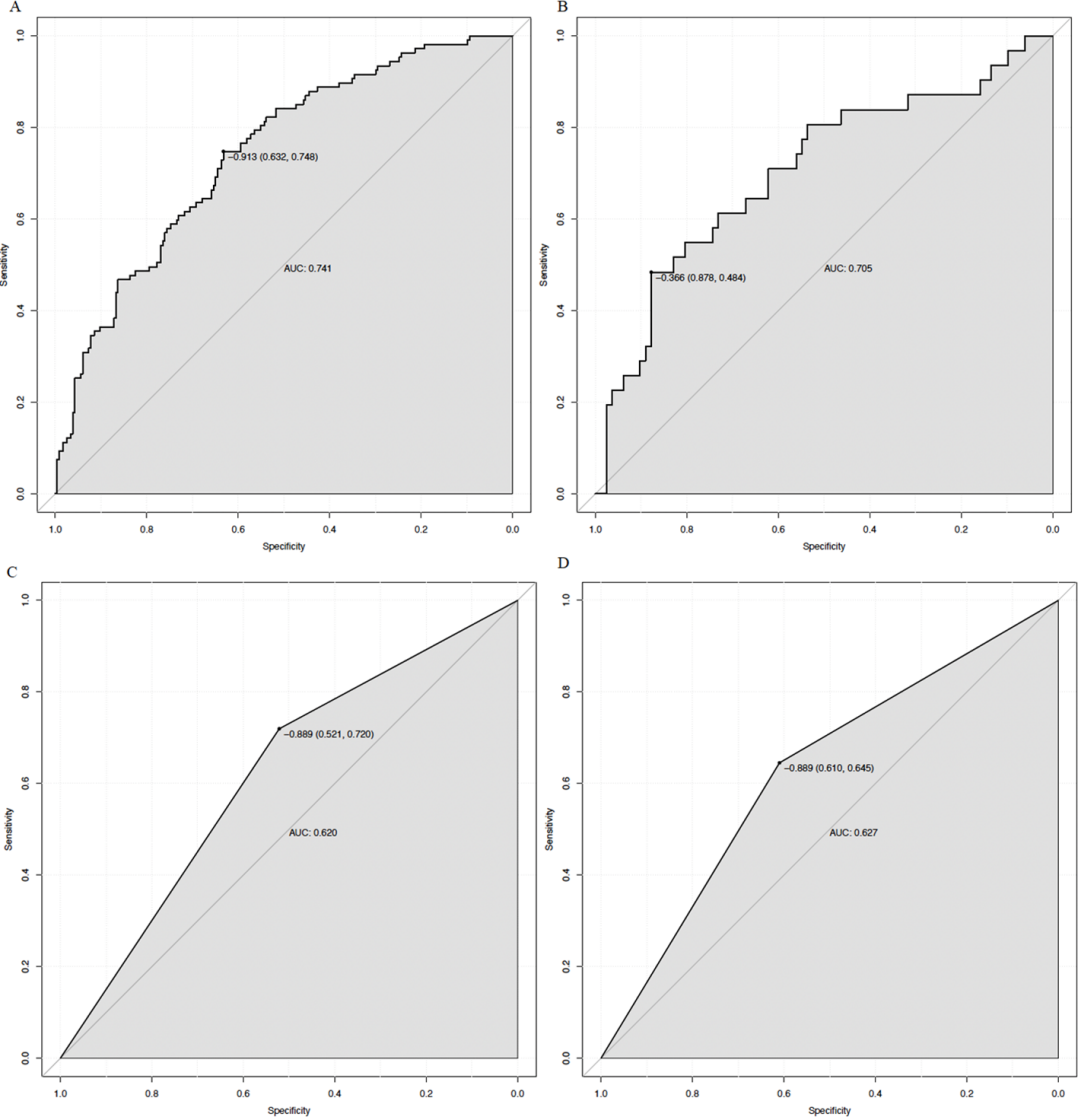
The ROC curve of the nomogram for predicting LNM in patient with breast cancer. The AUC of the nomogram for the prediction of LNM in the training set **(A)** and the validation set **(B)**. The AUC of the Ultrasonic lymph node status for the prediction of LNM in the training set **(C)** and the validation set **(D)**. Ultrasonic lymph node status (0 = normal, 1 = abnormal).

The ROC curve analysis showed that the model could differentiate between LNM and non-LNM patients with an AUC of 0.741 in the training cohort and 0.705 in the validation cohort, indicating acceptable discriminative performance (**Figures 3A, B**). According to common classification schemes for discriminative ability (e.g., Hosmer–Lemeshow: AUC 0.7–0.8 indicates acceptable discrimination), our model’s performance falls within the acceptable range. To further validate the clinical utility of our nomogram, we compared its predictive performance with that of the preoperative axillary ultrasound assessment performed by experienced breast radiologists. The Ultrasonic lymph node status (normal vs. abnormal) alone yielded an AUC of 0.620 in the training cohort and 0.627 in the validation cohort for predicting ALNM (**Figures 3C, D**). In comparison, our nomogram achieved significantly higher AUCs (training: 0.741, *P* = 0.0017; validation: 0.705, *P* = 0.1772; DeLong test). These results suggest that the nomogram provides improved discriminative ability over routine ultrasound assessment alone.

**Figure 4:**
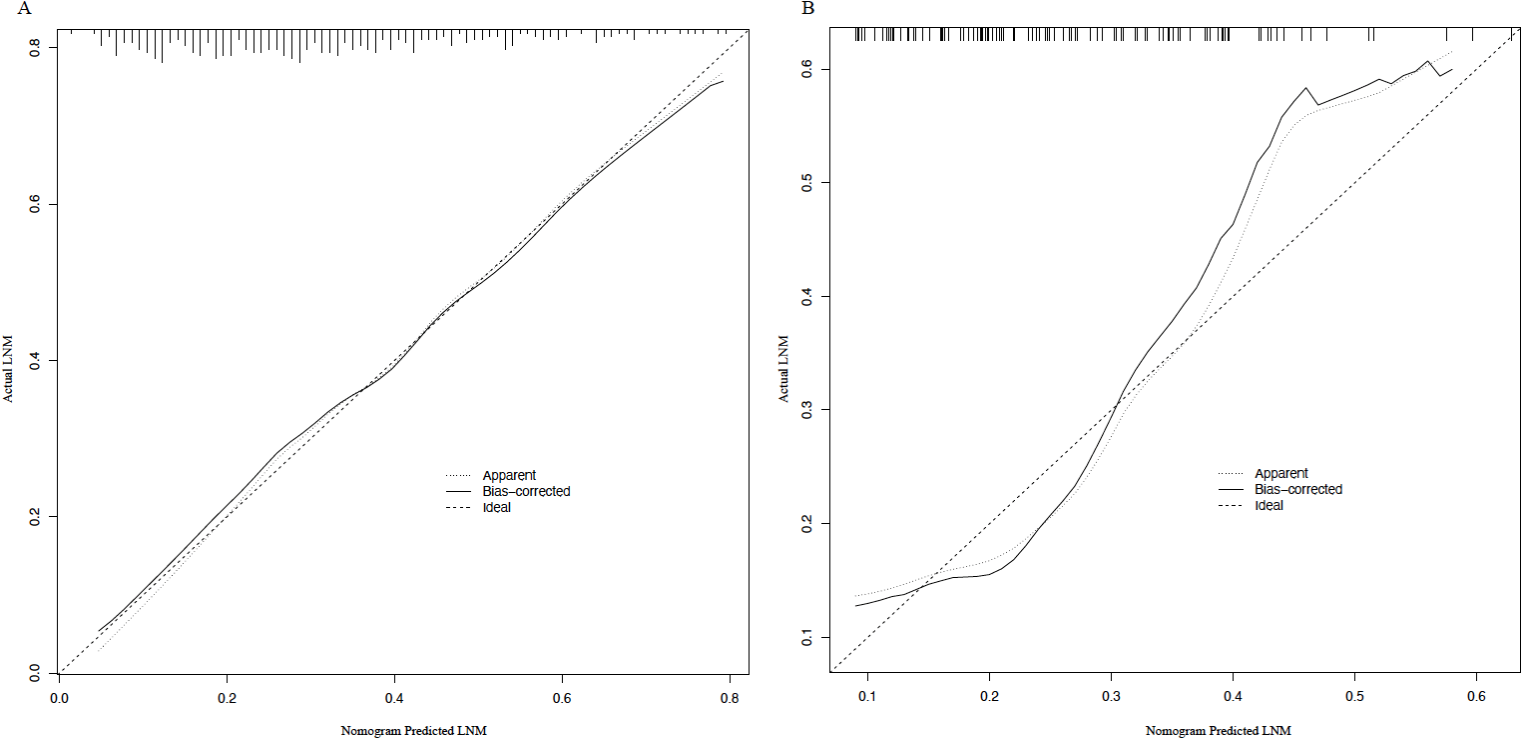
Calibration curves and DCA of the nomogram for predicting LNM. Calibration curves of the predicted nomogram in training set **(A)** and validation set **(B)**.

**Figure 5:**
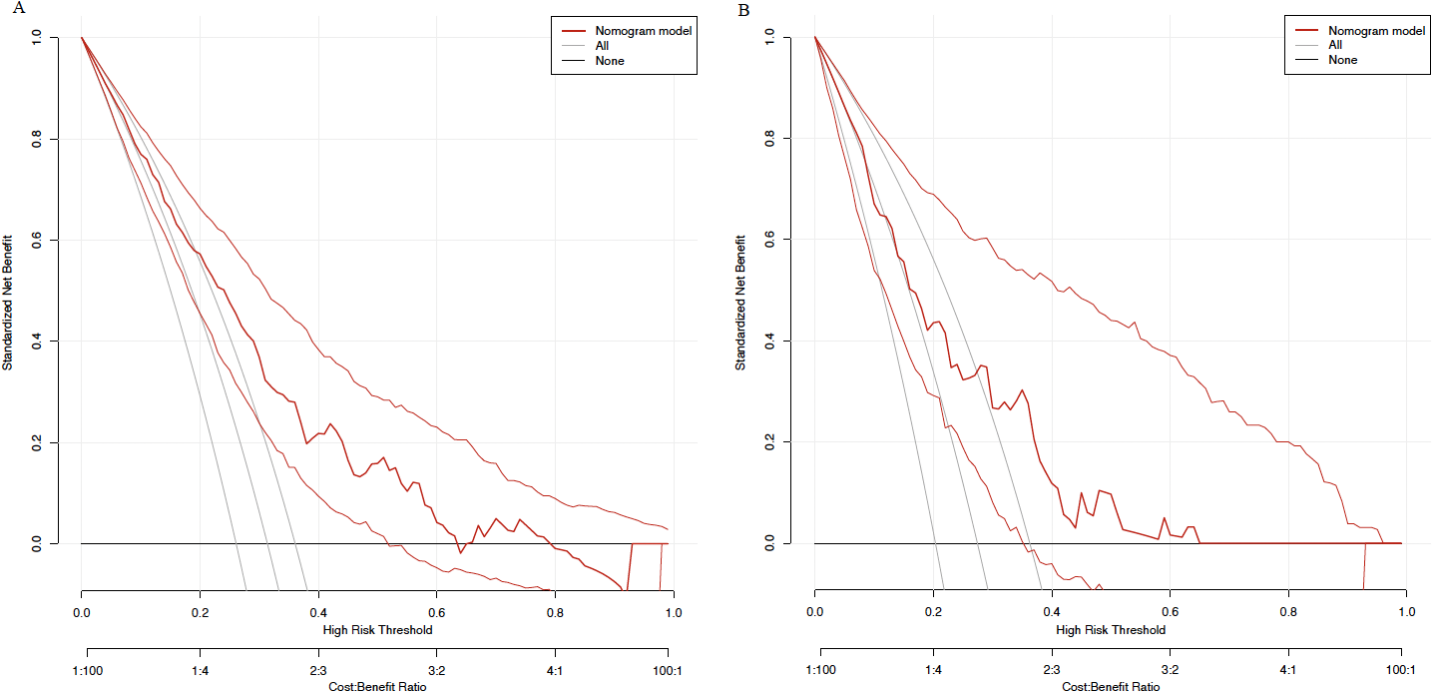
DCA of the nomogram in the training set **(A)** and the validation set **(B)**. DCA, decision curve analysis.

Additionally, the prediction probabilities generated by the model showed consistency with the observed outcomes in both the training set (**Figure 4A**) and the validation set (**Figure 4B**), indicating good calibration. The examination of the clinical impact curve (**Figures 5A, B**) demonstrates that the nomogram exhibits a substantial overall net benefit across a broad spectrum of threshold probabilities, suggesting a significant potential for clinical application.

## Discussion

Among patients with IBC, predicting ALNM is of paramount importance for clinical treatment decision-making. This study conducted an in-depth analysis of the clinicopathological characteristics, serological indicators, and ultrasound indices of the patients and discovered that there was a significant correlation between these factors and ALNM. Based on these findings, we selected key variables such as ER, Tumor size, circumscribe margins, Ultrasound Lymph node status, BI-RADS class and WBC and constructed a prediction model through multivariate regression analysis. Our results showed that in the training set and validation set, the proportion of IBC patients with axillary lymph node metastasis was 31.4% and 27.4%, respectively, which is consistent with reports in the existing literature (12). In addition, compared with MSKCC and SOC models, while the absolute discriminative performance (AUC 0.741 and 0.705) is considered acceptable for clinical decision support (17). Our nomogram demonstrated superior discriminative performance compared with ultrasound specialist assessment based on Ultrasonic lymph node status alone (AUC 0.741 vs. 0.620 in the training cohort). This improvement is likely attributable to the integration of additional predictors (tumor size, margin, ER, WBC, and BI-RADS) that capture complementary aspects of tumor biology. To our knowledge, this is one of the few nomograms that simultaneously incorporates ultrasound features, serological markers (including WBC), and pathological indicators in a Chinese population, while being explicitly designed to align with current axillary de−escalation strategies.

The paradigm of axillary surgery has been progressively narrowed by landmark de−escalation trials. ACOSOG Z0011 and AMAROS established that completion ALND can be safely omitted or replaced by axillary radiotherapy in selected patients with limited sentinel node involvement (18, 19). More recently, SOUND, INSEMA, and SENOMAC demonstrated that even SLNB can be safely omitted in cT1−2N0 patients with negative preoperative ultrasound, and that completion ALND is unnecessary for sentinel node macrometastases (6, 20, 21); a meta−analysis (CADONOT) confirmed a 65% lower lymphedema risk with SLNB alone versus ALND (6). The impetus for axillary de−escalation stems largely from the substantial morbidity associated with more extensive axillary surgery. ALND carries a significant risk of postoperative complications, including seroma formation (reported in 15–30% of patients), wound infections (5–10%), hematoma (3–5%), and prolonged drainage, which can delay adjuvant therapy and impair quality of life (18, 19, 22). In contrast, sentinel lymph node biopsy (SLNB) substantially reduces these risks: seroma rates drop to 5–10%, wound complications to 2–5%, and lymphedema to approximately 5–8% compared with 20–30% after ALND (7). Lymphedema, in particular, is a feared long−term sequela that can lead to chronic swelling, functional impairment, and recurrent infections. Landmark de−escalation trials have consistently demonstrated that omitting axillary surgery altogether in carefully selected patients (e.g., the SOUND and INSEMA trials) results in even lower rates of these complications without compromising oncologic outcomes (20, 21). Therefore, accurate preoperative identification of patients with a very low risk of ALNM is crucial to safely extend the benefits of avoiding any axillary surgery. Our nomogram is designed to support this precise risk stratification. Our nomogram directly addresses this clinical need by integrating clinicopathological, ultrasound, and serological indicators to predict ALNM risk preoperatively, thereby providing supportive information for preoperative risk stratification. However, due to its moderate discriminative ability, it cannot replace standard axillary staging or directly determine the omission of SLNB.

Beyond axillary staging, accurate preoperative assessment of primary breast tumors is equally critical for tailored surgical planning, particularly for non-palpable breast lesions which account for a significant proportion of screen-detected breast cancers. Precise localization of these lesions is essential to achieve negative surgical margins while minimizing the volume of healthy breast tissue resected, thereby improving cosmetic outcomes and patient quality of life. Traditional wire-guided localization, while widely used, has several limitations including wire migration, patient discomfort, and the need for preoperative radiological placement immediately before surgery. In recent years, advanced image-guided localization techniques have emerged as promising alternatives, offering enhanced precision and flexibility. Parisi et al. (23) compared radiofrequency (RF) localization alone with a combined RF and intraoperative ultrasound (IOUS) technique for non-palpable breast cancer, demonstrating that the combined approach resulted in significantly lower positive margin rates (11.1% vs. 27.8%) and reduced reoperation rates. Similarly, the same group reported their initial experience with the combined LOCalizer™ radiofrequency identification system and IOUS, which achieved 100% successful localization with no positive margins in their cohort of 25 patients (24). These studies highlight the value of integrating multiple imaging modalities and advanced localization technologies to optimize breast-conserving surgery. Our nomogram, which provides preoperative risk stratification for ALNM, complements these localization techniques by enabling comprehensive individualized surgical planning.

This study covers preoperative ultrasound imaging features commonly used in the clinic. Ultrasound technology is non-invasive, reproducible, and can be observed in real time, and has shown unique value in detecting small calcifications inside tumors and in imaging blood flow. In recent years, several clinicians have constructed models to predict axillary lymph node metastasis based on ultrasound features. Our study further optimizes and validates these existing models. Consistent with previous models, our results show that Tumor size, circumscribe margin features, BI-RADS classification, and Ultrasound lymph node status are independent predictors of ALNM in patients with IBC. Tumor size is the strongest predictor of ALN metastasis, a fact that has been demonstrated in multiple studies. A larger tumor volume means an increase in the number of tumor cells, which can lead to an increase in the complexity of the tumor microenvironment, thereby enhancing its metastasis potential (25). Circumscribe margins are key areas where tumors interact with surrounding normal tissue, and cells here tend to exhibit higher genomic instability, epithelial-mesenchymal transition (EMT) phenomena, and greater aggressiveness. By changing their morphology and movement patterns, these cells may play a key role in tumor invasion and metastasis, a finding that is consistent with previous observations by researchers (26–28).

The BI-RADS classification takes into account the morphological characteristics of the tumor, which are strongly associated with the risk of tumor metastasis (29). In addition, we integrated the positive indicators of ultrasound lymph node status assessment from previous studies, and patients meeting any of the following conditions were judged to have axillary ultrasound lymph node status abnormalities (30): (1) Round or oval lymph nodes were detected; (2) Hypoechoic nucleus or lymph node central portal disappeared; (3) The minimum diameter of lymph nodes is more than 5mm; 4. Irregular cortex or cortex thickness greater than 2mm. These changes are associated with modification of the lymph node microenvironment by tumor cells, including angiogenesis and recruitment of immune cells. In addition, Tumor morphology and CDFI were independent predictors of ALNM. Recent studies have shown that tumor morphology may be related to the remodeling of tumor extracellular mechanisms, and the high expression of mechanism metalloproteinases may lead to the degradation of extracellular matrix, thus promoting tumor invasion and morphological irregularity (31). The increased blood flow signal may be related to the inflammatory response in the tumor microenvironment, and the infiltration of inflammatory cells can release various cytokines, such as tumor necrosis factor α (TNF-α) and interleukin, which can promote blood vessel dilation and the formation of new blood vessels, making tumors more likely to metastasize (32).These ultrasound features incorporated into our model are readily available in all breast cancer patients, which guarantees the applicability and utility of our model.

In the clinical pathology parameters, our research findings indicate that ER is independent predictors of ALNM in IBC patients. As one of the key indicators in the diagnosis of breast cancer, ER has been widely confirmed to be closely related to the clinical outcome and prognosis of patients. ER status is not only an important parameter for evaluating tumor mutation load in breast cancer patients, but also its expression level is closely related to the expression of Ki67, PR and HER2, which has a significant impact on lymph node metastasis, tumor grade and lymphatic vessel invasion of invasive breast cancer (33). Recent studies have shown that ER promotes the growth and survival of invasive breast cancer cells by activating multiple signaling pathways, such as the PI3K/AKT/mTOR and MAPK/ERK pathways (34). Activation of these pathways enhances the aggressiveness and metastasis potential of tumor cells. In addition, estrogen can enhance the migration and invasion ability of breast cancer cells by regulating the expression of genes associated with tumor metastasis, such as promoting the expression of genes associated with epithelial stromal transformation (EMT) (35).

Existing research indicates that WBC counts are closely correlated with prognosis across multiple cancers. For instance, in colorectal cancer patients, elevated white blood cell counts correlate with poorer outcomes and an immunosuppressive tumor microenvironment (36). Additionally, white blood cell count is recognized as a key prognostic biomarker in non-smoking lung adenocarcinoma patients (37). In breast cancer, white blood cell count and its derived inflammatory markers, such as neutrophil-to-lymphocyte ratio (NLR) and platelet-to-lymphocyte ratio (PLR), have been extensively studied to evaluate their role in cancer prognosis (38). Interestingly, our multivariate analysis revealed that a higher WBC count was associated with a reduced risk of ALNM (OR = 0.802, 95% CI: 0.682–0.944). However, this finding should be interpreted with caution, as it is exploratory and derived from a retrospective single−center cohort. This protective effect may reflect competent systemic immune surveillance, where adequate circulating leukocytes facilitate the elimination of metastatic tumor cells in axillary lymph nodes. Alternatively, lower WBC counts in some patients might indicate poor nutritional status, bone marrow suppression, or advanced disease burden, all of which could correlate with higher metastatic potential. Nevertheless, we cannot exclude the possibility of residual confounding. Unmeasured factors – such as subclinical infections, concomitant medications (e.g., corticosteroids or anti−inflammatory drugs), systemic inflammatory states, or differences in disease stage – may influence both WBC count and ALNM risk. Reverse causality is also possible, whereby more advanced tumors might suppress bone marrow function or alter leukocyte trafficking. While some previous studies have reported that elevated WBC counts are associated with increased breast cancer risk, our findings suggest that the relationship between WBC and ALNM is complex and may depend on disease stage, patient population, and the balance between pro-tumor and anti-tumor immune responses (39). Given the exploratory nature of this finding, prospective studies with comprehensive covariate adjustment are needed to validate the independent protective association of WBC and to clarify the underlying mechanisms.

## Limitations

This study used a single-center retrospective design, and although we applied strict inclusion and exclusion criteria to ensure the accuracy of the data, selection bias is still a limitation that cannot be completely avoided. Furthermore, breast ultrasound is inherently operator dependent. A more specific limitation is that breast ultrasound is inherently operator dependent. In this study, all preoperative ultrasound evaluations were performed by a team of three experienced breast radiologists (each with more than 5 years of specialized experience) following a standardized scanning protocol. However, we did not formally assess inter-observer variability, which may affect the reproducibility of ultrasound-derived variables such as tumor margin, morphology, and axillary lymph node status. This represents an additional limitation of our study. However, we did not formally assess interobserver variability (e.g., using kappa statistics). This is a major limitation, as ultrasound variables such as tumor margin (circumscribe vs. vague), tumor morphology (regular vs. irregular), and axillary lymph node status (normal vs. abnormal) are subjective and may vary between operators. The lack of interobserver reproducibility assessment means that the performance of our nomogram when applied by different radiologists or in different institutions remains uncertain. Moreover, our model was developed and internally validated only in a single-center retrospective Chinese cohort. External validation in independent, preferably multicenter and multinational, cohorts is essential to assess its reproducibility across different populations (e.g., Western or other Asian populations), ultrasound operators, and healthcare settings. The current model’s performance in other surgical environments remains uncertain. In order to enhance the universality and reliability of the study results, future studies should adopt a multicenter, randomized controlled, large sample size prospective study design to further validate the findings of this study. Prospective studies should also include rigorous training and quality control measures for ultrasound operators, and report inter-observer agreement (e.g., kappa statistics) to ensure the generalizability of predictive models.

## Conclusion

In the era of axillary surgical de−escalation, accurately identifying patients with a sufficiently low risk of axillary lymph node metastasis is of paramount clinical importance. Our nomogram offers several practical advantages: it integrates three readily available data categories (clinicopathological, ultrasound, and serological indicators), uses only routine preoperative tests without requiring advanced imaging or genetic profiling, and is specifically developed and validated in a Chinese cohort. With acceptable discriminative performance (AUC = 0.741 in the training cohort, 0.705 in the validation cohort). However, given its moderate predictive accuracy, this model cannot directly determine whether sentinel lymph node biopsy (SLNB) may be omitted, nor can it replace standard axillary staging. At present, the nomogram should be viewed only as auxiliary support for preoperative risk stratification — for example, to identify patients with a higher probability of metastasis who may warrant more rigorous preoperative evaluation. Decisions to omit axillary surgery must continue to follow established trial criteria (e.g., SOUND, INSEMA) and current guideline recommendations. Prospective, multicenter external validation is necessary before this nomogram can be considered for routine clinical implementation.

## Data Availability

The raw data supporting the conclusions of this article will be made available by the authors, without undue reservation.
